# Schistosomiasis Mansoni: Novel Chemotherapy Using a Cysteine Protease Inhibitor

**DOI:** 10.1371/journal.pmed.0040014

**Published:** 2007-01-09

**Authors:** Maha-Hamadien Abdulla, Kee-Chong Lim, Mohammed Sajid, James H McKerrow, Conor R Caffrey

**Affiliations:** Sandler Center for Basic Research in Parasitic Diseases, California Institute for Quantitative Biomedical Research, University of California San Francisco, San Francisco, California, United States of America; St. George's, University of London, United Kingdom

## Abstract

**Background:**

Schistosomiasis is a chronic, debilitating parasitic disease infecting more than 200 million people and is second only to malaria in terms of public health importance. Due to the lack of a vaccine, patient therapy is heavily reliant on chemotherapy with praziquantel as the World Health Organization–recommended drug, but concerns over drug resistance encourage the search for new drug leads.

**Methods and Findings:**

The efficacy of the vinyl sulfone cysteine protease inhibitor K11777 was tested in the murine model of schistosomiasis mansoni. Disease parameters measured were worm and egg burdens, and organ pathology including hepato- and splenomegaly, presence of parasite egg–induced granulomas in the liver, and levels of circulating alanine aminotransferase activity as a marker of hepatocellular function. K11777 (25 mg/kg twice daily [BID]), administered intraperitoneally at the time of parasite migration through the skin and lungs (days 1–14 postinfection [p.i.]), resulted in parasitologic cure (elimination of parasite eggs) in five of seven cases and a resolution of other disease parameters. K11777 (50 mg/kg BID), administered at the commencement of egg-laying by mature parasites (days 30–37 p.i.), reduced worm and egg burdens, and ameliorated organ pathology. Using protease class-specific substrates and active-site labeling, one molecular target of K11777 was identified as the gut-associated cathepsin B1 cysteine protease, although other cysteine protease targets are not excluded. In rodents, dogs, and primates, K11777 is nonmutagenic with satisfactory safety and pharmacokinetic profiles.

**Conclusions:**

The significant reduction in parasite burden and pathology by this vinyl sulfone cysteine protease inhibitor validates schistosome cysteine proteases as drug targets and offers the potential of a new direction for chemotherapy of human schistosomiasis.

## Abstract 2

A significant reduction in parasite burden and pathology by a vinyl sulfone cysteine protease inhibitor suggests a new direction for chemotherapy of human schistosomiasis.

## Editors' Summary

**Background.:**

Schistosomiasis, a disease caused by a type of parasitic flatworm that lives in the blood, infects around 200 million people worldwide. The disease is a serious problem in sub-Saharan Africa, South America, China, and southeast Asia. Although this disease can kill, it is better known as a lifelong chronic infection with debilitating symptoms mainly due to an immune reaction raised against parasite eggs trapped in the liver, spleen, and gut. The worm's life cycle is complicated and involves a free-swimming form that emerges from certain types of snails that live in lakes and ponds. This can penetrate the skin of people in contact with the water. After a period spent in the skin and around the lungs, the parasites move to veins around the gut, and develop into adult worms that mate and lay eggs. These eggs eventually return to the water through the person's feces or urine. A particular group of proteins called cysteine proteases are thought to be very important in the biology of these worms, especially in their function as digestive enzymes in the parasite's gut. These proteases could represent an exciting opportunity for development of new drugs to treat schistosomiasis. The researchers are looking at whether it is possible to block the activity of cysteine proteases and, as a result, kill the worms or prevent them from developing and thriving.

**Why Was This Study Done?:**

At the moment there is only one drug, praziquantel, in common use for treatment of schistosomiasis; it is cheap and effective. However many organizations are worried about relying on a single drug to treat a serious disease which affects so many people worldwide. The research group here has been looking at molecules that block cysteine protease activity, to see if any of these could be good drug candidates for schistosomiasis. One molecule they have been looking at goes by the name of K11777, which is under evaluation as a drug candidate for another parasitic infection (Chagas' disease). Here, the researchers wanted to find out whether K11777 had any activity against schistosome worms.

**What Did the Researchers Do and Find?:**

In this study, the researchers deliberately infected laboratory mice with the schistosome parasite. These mice were then either injected with K11777 solution twice daily, or with equivalent volumes of water as a comparison. The researchers examined the effects of injecting K11777 either “early” in infection (using a 14 day course, starting 1 day after infection with the parasite) or “late” in the worms' development (using an 8 day treatment course starting 30 days after infection). The outcomes used as measures of success of treatment with K11777 included the number of worms recovered from mice after euthanasia, the number of worm eggs counted in the liver; the extent of the damage to the liver; and finally, the researchers also looked at activity levels of cysteine proteases in the worms themselves, in particular, those proteases associated with the parasite gut.

The results of the early-treatment experiment showed a substantial decrease in worm numbers and egg production. In five of the seven mice treated, eggs were eliminated entirely. Also, there was little measurable liver damage. For the late-treatment experiment, decreased burdens of worms and eggs in the livers of K11777 treated mice were also found, and there was less damage to the livers. Those worms surviving treatment and removed from mice also had much less activity of gut cysteine proteases suggesting that K11777 exerts its effects by targeting worm cysteine proteases.

**What Do These Findings Mean?:**

These experiments show that K11777 is a potent antischistosomal agent in mice. It might therefore be a good ‘candidate' molecule for developing future treatments for human schistosomiasis. However, before that stage can be reached, it would be important to carry out clinical trials to test whether K11777 is both safe and effective in schistosomiasis patients. Full details as to which worm cysteine protease(s) is the critical target of K11777 would also need to be worked out, and more information would be needed as to whether the dosing plan used in this study (twice-daily injections for a week to 14 days) can be decreased.

**Additional Information.:**

Please access these Web sites via the online version of this summary at http://dx.doi.org/10.1371/journal.pmed.0040014.

- World Health Organization pages about schistosomiasis including links to details on further research into the disease
- Information from the US Centers for Disease Control for patients and health professionals about schistosomiasis
- Wikipedia pages on schistosomiasis (Wikipedia is an internet encyclopedia anyone can edit)
- PLoS Neglected Tropical Diseases is a new journal from the Public Library of Science that is devoted to publishing research on the world's most neglected tropical diseases, including schistosomiasis

## Introduction

Schistosomiasis, or bilharzia, is a major public health problem in tropical and subtropical regions including sub-Saharan Africa, South America, China, and Southeast Asia. An estimated 200 million people are infected, and a total of 400 million are at risk. Caused by blood flukes of the genus *Schistosoma,* the disease is associated with a chronic and debilitating morbidity manifested by sequelae such as cognitive impairment, lassitude, and growth stunting [1]. Disease pathology is primarily due to an immune-mediated granulomatous response to parasite eggs trapped in the liver, spleen, and other peritoneal organs [2]. The World Health Organization recommends a strategy of disease control such that the consequences of chronic infection are reduced and maintained at a level that is no longer considered a public health burden [1].

Up to 1975, chemotherapy of schistosomiasis had relied on the use of antimonials and a variety of other drugs targeting DNA synthesis, and carbohydrate and protein metabolism [3]. Since then, three drugs have been used for treatment: metrifonate, oxamniquine, and praziquantel (PZQ); however, it is the latter that is now universally employed, as recommended by the World Health Organization for either individual or mass treatment [4]. PZQ is effective against all five species of schistosomes infecting humans: *S. mansoni, S. haematobium, S. japonicum, S. intercalatum,* and *S. mekongi*. Juvenile parasites (between 7 and 28 days-old), however, are less susceptible to PZQ than adults ([5,6] and references therein), so individuals continuously exposed to new infections must be retreated. PZQ is well tolerated, easily administered in tablet form, and inexpensive, at around US$0.30–0.60 per 600 mg dose [1,7].

Despite the success of PZQ, the prospect of relying on a single drug to treat 200 million people is of concern [8] and the potential for drug resistance, particularly in areas of high transmission, must be considered. Suspicions were raised that low cure rates for PZQ in a schistosomiasis mansoni outbreak in Senegal in the early 1990s were partly due to drug resistance, although alternative interpretations discussed included the high pretreatment levels of infection and intense disease transmission that resulted in individuals harboring large numbers of immature parasites inherently less sensitive to the drug [9]. In another study, resistant isolates of S. mansoni had been obtained from patients multiply treated with PZQ in the Nile Delta. These parasites showed 2- to 6-fold elevated ED_50_ (the dose required to kill 50% of parasites) values compared to isolates not previously exposed to PZQ [10]. Finally, resistance to PZQ has been selected for in laboratory mice [11]. Whether or not the identified or suspected resistance turns out to be clinically relevant, there are sufficient grounds for both continued vigilance and the development of alternative chemotherapies.

One encouraging approach to novel antiparasitic agents is the development of small-molecule inhibitors of cysteine proteases (CPs) [12]. CPs are fundamental to the metabolism of many parasites [13], and CP inhibitors have been shown to kill protozoan parasites in both culture [14,15] and animal models of disease [16,17]. Schistosomes also express a number of CPs that function in digestion, reproduction, and protein turnover [18], and it has been shown that fluoromethyl ketone-based CP inhibitors decrease worm and egg burdens in mice infected with S. mansoni [19]. Since these studies were published, significant advances—such as improved solubility and bioavailability, and reduced toxicity—have been incorporated into the design of new-generation CP inhibitors. One such inhibitor, *N*-methyl-piperazine-phenylalanyl-homophenylalanyl-vinylsulfone phenyl (K11777), is a well-tolerated, orally bioavailable compound currently in the late stages of preclinical development for Chagas' disease (caused by infection with a protozoan parasite, Trypanosoma cruzi) [20,21]. For this report, we assessed the efficacy of K11777-treatment in mice infected with S. mansoni by measuring worm and egg burdens, and liver and spleen pathologies. Also, identification of the protease target(s) of K11777 in the parasite was sought.

## Methods

### Maintenance of the S. mansoni Life Cycle

A Puerto Rican isolate of S. mansoni was maintained in the laboratory using Biomphalaria glabrata snails and mice as intermediate and definitive hosts, respectively [22]. BALB/C mice (aged 5–6 wk) were purchased from Charles River (http://www.criver.com) and maintained at the Animal Care Facility, Veterans Affairs Medical Center (VAMC) San Francisco in accordance with protocols approved by the VAMC Institutional Animal Care and Use Committee. Infections of mice with *S. mansoni* were initiated by subcutaneous injection of 150 cercariae (infective larvae). At 49 d postinfection (p.i.), mice were euthanized with an intraperitoneal (i.p.) injection of 0.05 mg/g sodium pentobarbital, and adult worms were perfused [23] in RPMI 1640 medium (Invitrogen, http://www.invitrogen.com) containing 10% bovine calf serum (Invitrogen).

### Drug Treatment

**Figure 1:**
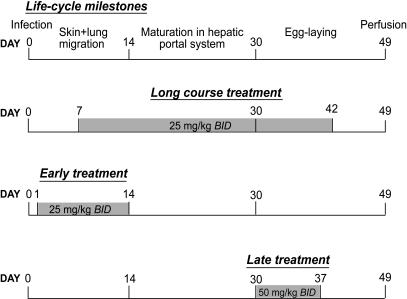
Experimental Design for Assessing the Therapeutic Effect of the Cysteine Protease Inhibitor K11777 on Mice Infected with S. mansoni All mice were infected with 150 S. mansoni cercariae injected subcutaneously. K11777 was administered intraperitoneally in 100 μl of water at the dosages and timings indicated. Control mice received water alone. All mice were perfused at 49 days p.i., and worm and egg burdens and organ pathology were assessed.

K11777 was dissolved in sterile distilled water at a concentration of 5 or 10 mg/ml and administered in 100 μl by i.p. injections twice daily (BID) according to the schedules indicated in Figure 1. Infected control mice were administered an equal volume of water. Apart from the long-course experiment, two early and two late drug treatments were conducted with five mice per experiment.

### Measurement of Total Worm Burden, Hepatic Egg Burden, and Organ Weights

Numbers of male and female worms obtained by perfusion were recorded, as were the weights of livers and spleens. For determination of parasite egg burdens, livers from infected animals were digested in 0.7% porcine trypsin in PBS for 1 h at 37 °C on an orbital shaker. Eggs were then sedimented at 4 °C and counted under a dissecting microscope as described previously [24].

### Measurement of Hepatic Pathology

As a quantitative indicator of hepatocellular damage, circulating levels of alanine aminotransferase (ALT) were measured. Heparinized blood samples were obtained by cardiac puncture at the time of euthanasia and ALT activity measured in a Beckman Chemical Analyzer. Baseline plasma ALT levels were also determined for age-matched uninfected animals. For histopathology, a piece of one liver lobe was removed and fixed in 4% PBS-buffered formaldehyde. The tissue was paraffin embedded, sectioned at 5 μm, and processed for hematoxylin and eosin staining using standard protocols.

### Measurement of S. mansoni Cysteine Protease Activity

Upon perfusion, worms were washed five times in RPMI-1640 without serum and frozen at −80 °C. Soluble worm extracts were prepared and endogenous CP activity measured essentially as previously described [25] using benzyloxycarbonyl-phenylalanyl-arginyl-7-amido-4-methylcoumarin (Z-Phe-Arg-AMC; Bachem, http://www.bachem.com) and Z-Arg-Arg-AMC as substrates for cathepsins B and L, and cathepsin B, respectively. Reaction of worm extracts with the radiolabeled CP inhibitor ^125^I-DCG-04 was carried out as previously described [26]. Prior to labeling, some samples were preincubated for 1 h with the selective cathepsin B inhibitor CA-074 [27] at a final concentration of 5 μM. All samples were heated to 70 °C for 10 min in 1× NuPAGE LDS sample buffer (Invitrogen) containing 50 mM DTT, and were subjected to SDS-PAGE through 4%–20% gradient gels. Labeled proteins were visualized by autoradiography using standard methodologies.

### Statistics

The data from each early or late experiment were combined for presentation and statistical analysis. Data were plotted as individual points and included median values. All data were subjected to the Mann-Whitney nonparametric test to determine any statistical differences in egg and worm burdens, and organ pathologies between treated and untreated control mice.

## Results

### K11777 Significantly Decreases S. mansoni Egg and Worm Burdens, and Hepatosplenomegaly

An initial range-finding experiment (“long course,” Figure 1) was begun on day 7 p.i. and continued for 35 d with i.p. injections of K11777 at 25 mg/kg BID. By day 7 p.i., immature schistosomula (larvae) have left the skin invasion site, attained the venous system, and are in transit through the lung capillaries before finally settling in the hepatic portal venous system. The length of time required for this migration depends on the species of schistosome, but for S. mansoni it is nominally 14 d [28,29]. By day 49 p.i., at the time of worm recovery, male and female worms have matured and paired, and eggs are found in the liver, intestine, and feces [30].

**Figure 2:**
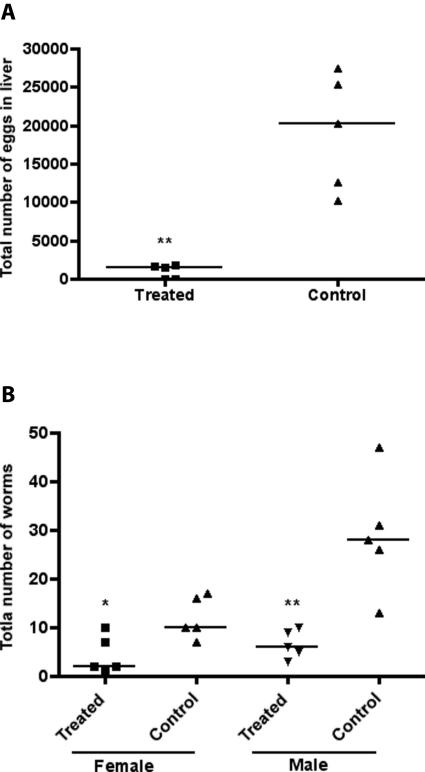
Long-Course Treatment with K11777 Dramatically Decreases Egg and Worm Burdens in Mice Infected with S. mansoni Results from the experiment outlined in Figure 1 are shown for egg (A) and worm (B) burdens. Points represent data from individual treated or untreated (control) infected mice. The horizontal bars represent median values: *p = 0.03; **p = 0.008.

**Figure 3:**
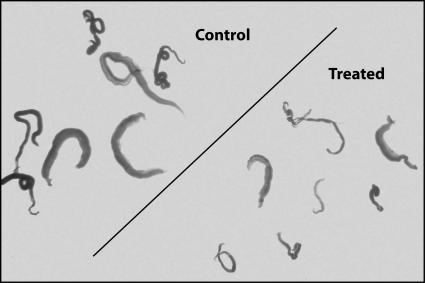
Long-Course Treatment with K11777 of Mice Infected with S. mansoni Retards Worm Growth Representative worms treated with K11777 in the experiment outlined in Figure 1 are shown.

As shown in Figure 2A, K11777 dramatically decreased (by 92%) the total number of eggs recovered in livers of treated mice (median of 1,538 eggs/liver; *n* = 5) compared to untreated controls (median of 20,210 eggs/liver; *n* = 5). Egg production is contingent on worm maturation, pairing of males and females, and the support of the metabolic needs of the female. K11777 clearly disrupted this developmental process by directly killing worms: female and male burdens were reduced by 80% and 79%, respectively (Figure 2B). Furthermore, those surviving worms exposed to K11777 were clearly smaller in both girth and length (Figure 3).

**Figure 4:**
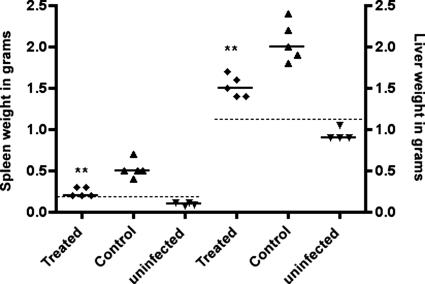
Long-Course Treatment with K11777 Resolves Egg-Induced Hepato- and Splenomegalies in S. mansoni–Infected Mice Points represent data from individual mice that were infected and treated, infected and untreated (control), or uninfected (Figure 1). The horizontal bars represent median values: ^**^ *p =* 0.007*.* The hatched lines represent the baseline for age-matched uninfected mice.

The pathology of chronic schistosomiasis is due to a granulomatous inflammation in response to eggs that are trapped in host tissues, particularly the liver and spleen in the case of S. mansoni [2]. As a consequence, gross hepato- and splenomegaly become obvious in infected animals, and punctate fibrotic foci are visible on the surface of the liver after necropsy. With treated mice, few fibrotic lesions were visible on the liver (unpublished data), and spleen and liver weights were decreased by 54% and 29%, respectively, compared to untreated controls (Figure 4).

### Treatment with K11777 Targets S. mansoni Cathepsin B1

**Figure 5:**
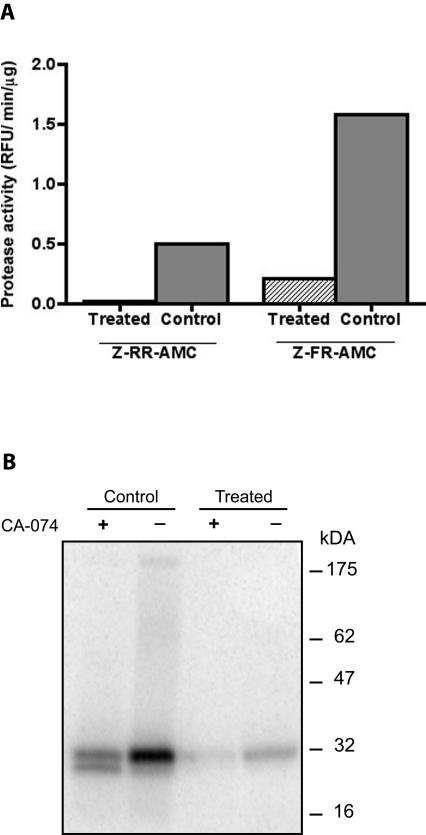
A Principal Molecular Target of K11777 in S. mansoni is the Cathepsin B1 Cysteine Protease (A) Extracts of worms exposed to long-course treatment with K11777 (see Figure 1) had significantly less protease activity (relative fluorescence units/min/μg of extract) against the cathepsin B–selective substrate Z-Arg-Arg-AMC (Z-RR-AMC) or the cathepsins B- and L-specific substrate Z-Phe-Arg-AMC (Z-FR-AMC). (B) By SDS-PAGE, a major 31 kDa protease species in extracts of control worms (lane 2) or worms exposed to K11777 (lane 4) reacted with the radiolabeled CP inhibitor ^125^I-DCG-04. The reaction was approximately 10-fold less intense in the latter by scanning densitometry. For both extracts, the reaction with ^125^I-DCG-04 could be inhibited by prior incubation with the cathepsin B–selective inhibitor CA-074 (lanes 1 and 3), thus confirming the 31 kDa reactive species as cathepsin B1 [31]. The second, faster-migrating protease species in lane 1 is consistent with a schistosome cathepsin L [31].

To determine the molecular target(s) of K11777, extracts were prepared from worms surviving the long-course treatment and processed for endogenous proteolytic activity. Substantially less CP activity (per microgram of protein) was measured in these extracts compared to those of control worms (Figure 5A). Activity against the selective cathepsin B substrate Z-Arg-Arg-AMC, or the substrate for cathepsins B and L Z-Phe-Arg-AMC, was decreased by approximately 90%. Upon incubation of control untreated worm extracts with the specific CP active site “tag,” ^125^I-DCG-04, a major protease species at 31 kDa was labeled after resolution by SDS-PAGE (Figure 5B, lane 2). By contrast, using the same protein concentration of worms exposed to K11777, active site labeling was decreased by approximately 10-fold (by scanning densitometry; Figure 5B, lane 4). For extracts of both control and drug-exposed worms, labeling with ^125^I-DCG-04 could be inhibited by prior incubation with the cathepsin B-selective inhibitor CA-074 (Figure 5B, lanes 1 and 3, respectively), confirming the 31 kDa reactive species as the gut-associated cathepsin B1, in accordance with previous data [31]. The second and faster migrating protease species in lane 1 is consistent with a schistosome cathepsin L [31].

### “Early” Treatment with K11777 Eliminates Egg Production and Severely Impacts Worm Burden

**Figure 6:**
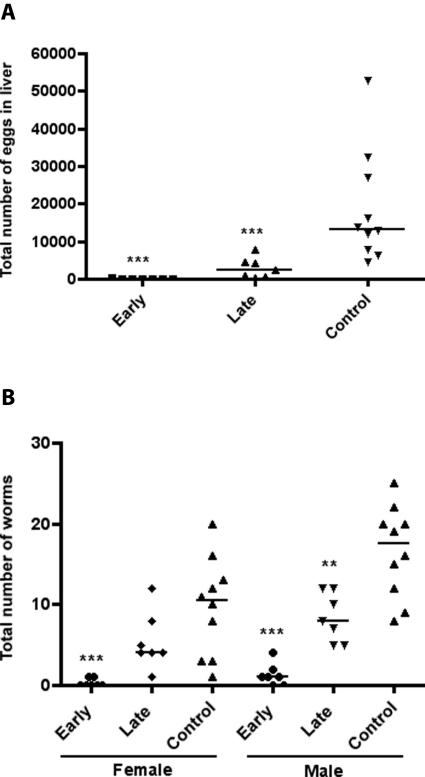
Early and Late Treatment of Mice with K11777 Significantly Decreases Egg and Worm Burdens in Mice Infected with S. mansoni Results for egg (A) and worm (B) burdens following the experimental protocol depicted in Figure 1 are shown. Points represent data from individual mice which were combined from either two early or two late treatment experiments. The horizontal bars represent median values: **p = 0.002, *** *p* = 0.0007. The data comparing female worm burdens between late treatment and untreated controls were not statistically different (*p* = 0.2).

A second treatment, termed “early” and designed to coincide directly with the skin and lung migratory phases of the parasite (Figure 1), was started at day 1 p.i. and continued for 14 d with 25 mg/kg BID. This drug regimen resulted in parasitologic cure (elimination of egg production) in five of the seven mice tested (Figure 6A). The remaining two mice each produced just 454 and 159 eggs/liver. By contrast, livers from untreated control mice (*n* = 10) had a median value of 13,376 eggs/liver. The same drug regimen dramatically reduced female and male worm survival by 90% and 88%, respectively, and in the five mice without parasite eggs in the liver, no female worms were recovered (Figure 6B). As found for the long-course drug regimen, the few worms surviving treatment were considerably smaller (unpublished data).

### Early Treatment with K11777 Resolves Pathology in the Liver and Spleen

**Figure 7:**
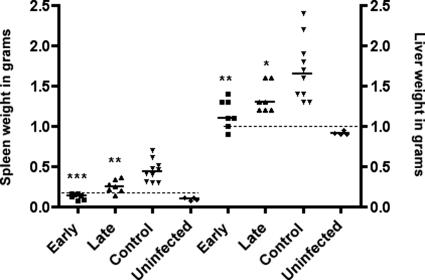
Early and Late Treatment of Mice with K11777 Decreases Hepato- and Splenomegalies in Mice Infected with S. mansoni Points represent data from individual mice which were combined from either two early or two late treatment experiments (see Figure 1). The horizontal bars represent median values: * *p* = 0.02, ** *p* = 0.004, ***p = 0.0003. The hatched lines represent the baseline for age-matched uninfected mice.

**Figure 8:**
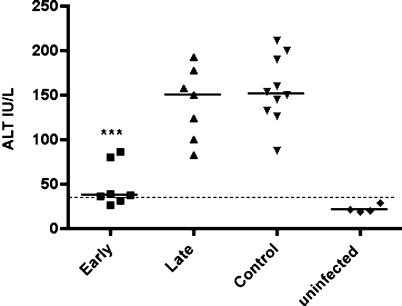
Early Treatment of S. mansoni–Infected Mice with K11777 Retards Hepatocellular Damage Arising from Parasite Eggs Plasma samples from mice in experiments outlined in Figure 1 were quantitatively assayed for the presence of ALT activity as a marker for hepatocellular damage. Points represent data from individual mice which were combined from either two early or late treatment experiments. The horizontal bars represent median values; ***p = 0.002. For plasma from late-treatment mice, ALT activity was not significantly different (*p* = 0.9) from untreated controls, most likely due to the re-emergence of hepatocellular toxicity upon cessation of treatment at day 37 p.i.—i.e., 12 days prior to the end of the experiment and recovery of worms. The hatched line represents the baseline for age-matched uninfected mice.

**Figure 9:**
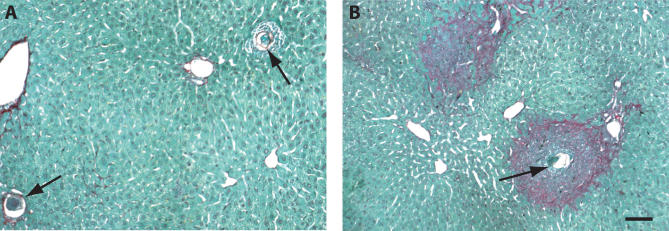
Late Treatment of *S. mansoni–*Infected Mice with K11777 Decreases the Granulomatous Reaction to Parasite Eggs Trapped in the Liver After necropsy of mice from experiments outlined in Figure 1, livers were sectioned at 5 μm and stained with hematoxylin and eosin. Arrows in representative sections indicate schistosome eggs; the granulomatous reaction to the eggs is much reduced after the late-treatment regimen (A), compared to the intense reaction visible in sections from untreated control mice (B). Note that after early K11777 treatment, eggs or egg-associated granulomas were not found (unpublished data). Scale bar = 100 μm.

Early treatment with K11777 decreased spleno- and hepatomegalies by 68% and 33%, respectively, relative to the untreated controls (Figure 7). For both organs, weights were similar to those from age-matched uninfected mice (*n* = 4). Liver function in treated animals, as judged by median plasma levels of ALT, was close to that of uninfected mice (38 versus 21 IU/l), whereas the ALT level in untreated control mice was approximately four times higher (152 IU/l; Figure 8). Gross evidence of fibrotic foci on the liver surface at necropsy was absent in treated mice (unpublished data), and this resolution in pathology associated with parasite eggs was confirmed by light microscopy. Indeed, the very low egg burdens of 454 and 159 eggs/liver in two of the seven livers of treated mice made difficult the identification of any granulomas. By contrast, many large granulomas were found in liver sections from untreated controls (Figure 9B), each consisting of a dense cellular infiltrate that included macrophages, eosinophils, and lymphocytes.

### “Late” Treatment with K11777 Significantly Reduces Egg Production and Worm Burden

By 30 days p.i., worms have matured, paired and commenced egg-production in the portal system of the host. Accordingly, a “late” treatment was initiated to coincide with this life-cycle phase (Figure 1). K11777 at 50 mg/kg BID for 8 d decreased egg burdens by 81 % (median of 2,545 eggs/liver; *n* = 7) compared to untreated controls (Figure 6A). With respect to worm burdens (Figure 6B), the 54 % reduction in the median value for females was not significantly different from the untreated control group (*p* = 0.2), whereas the 57% reduction for male worms was (*p* = 0.0007). As with the two previous drug treatments, worms exposed to K11777 were noticeably smaller (unpublished data).

### Late Treatment with K11777 Decreases Liver and Spleen Pathology

Median spleen and liver weights of treated mice were decreased by 46% and 23%, respectively, compared to untreated controls (Figure 7). Also, fibrotic foci on the surface of the livers were less numerous (unpublished data). However, liver function, as measured by assay of plasma ALT (median of 150 IU/l), was not improved over that of untreated control mice (*p* = 0.9; Figure 8). By light microscopy, egg-associated granulomas in treated mice were poorly developed (Figure 9A) compared to the extensive granulomatous reaction surrounding parasite eggs in livers from untreated control mice (Figure 9B).

## Discussion

In this study we have demonstrated that K11777 is a potent schistosomicide that achieves parasitologic cure (absence of eggs) and retards egg-associated organ pathology when given early in infection or significantly decreases egg burden and organ pathology when given late in infection. Furthermore, using CP-specific substrates and active site labeling, we identified one molecular target of K11777 as the major CP associated with the schistosome gut, cathepsin B1.

PZQ is the recommended drug for treatment of schistosomiasis; however, concerns about reliance on a single drug to treat 200 million people, and the potential for drug resistance, make identification of alternative schistosomiasis chemotherapies a high priority. Over the last ten years, an intensive medicinal chemistry effort has concentrated on the design of small-molecule inhibitors of CPs as novel antiparasitic agents, including against falciparum malaria [14], Chagas' disease (T. cruzi [16]) and African sleeping sickness (T. brucei [17]). The molecular targets are the Clan CA (papain-like) CPs that are ubiquitous in parasitic protozoa [13] and helminths [18,32].

Previously developed CP inhibitor scaffolds using dipeptidyl diazo- and fluoromethyl ketones had antitrypanosomal [17] and antischistosomal [19] activities. However, concerns about diazo teratogenicity [33] and fluoride toxicity [34] dissuaded their continued development. Further medicinal chemistry exploration identified the peptidomimetic vinyl sulfones like K11777 as improved inhibitor leads because they are nontoxic, relatively unreactive with glutathione, and stable in water [35].

K11777 has undergone extensive pharmacokinetic [21,36] and toxicological screening as a potential therapy for Chagas' disease. The inhibitor is water soluble, orally bioavailable (20%), and, at the highest therapeutic dose of 50 mg/kg BID used here, neither toxic nor mutagenic (Table 1). At the same dosage, K11777 cures T. cruzi infection in mice via inhibition of the CP cruzain [16]. The compound originated from an industrial medicinal chemistry program to produce inhibitors of human cathepsins for use in vivo [37,38], and one concern about its use as an antiparasitic is its potential to inhibit host orthologous proteases. Although K11777 is indeed a potent inhibitor of human cathepsin S in vitro, evidence that it could significantly decrease host protease activity in vivo was not found (J. Palmer, personal communication). Also, for practical purposes, only short-course disease therapies are envisaged, and both the present compound (Table 1, see also [16]) and other vinyl sulfones [39], demonstrate satisfactory toxicological profiles in this setting.

**Table 1:**
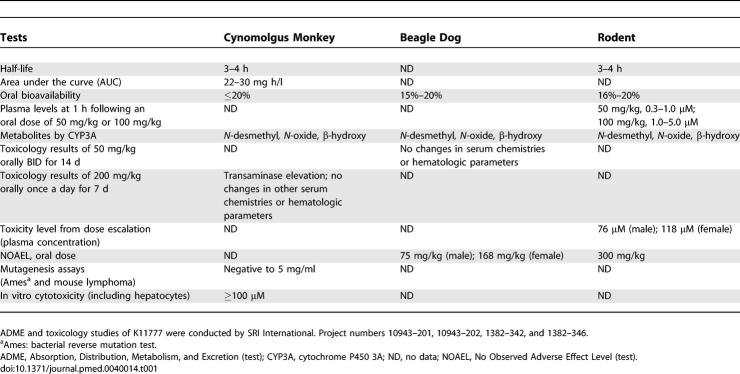
Summary of ADME and Toxicology of K11777

With respect to *Schistosoma,* juvenile and adult worms express a number of Clan CA proteases, including cathepsins B1, F, and L [18,31,32], which, in the parasite gut, operate synergistically and with some functional redundancy to degrade host proteins as a source of nutrition [40]. As yet, the issue of whether any one molecule represents an appropriate drug target is unclear [18] and given the lack of selectivity of K11777 for any single Clan CA protease [37], it is quite possible that our data arise from the inhibition of more than one schistosome protease target. Nonetheless, it is clear that K11777 demonstrates significant schistosomicidal activity, including parasitologic cure, and that one molecular target of the compound is the major gut-associated cathepsin B1 [18,25,31].

An initial range-finding drug trial (“long-course,” starting at day 7 p.i. for 35 d [Figure 1]) established K11777 as a potent schistosomicide by dramatically reducing both egg and worm burdens. Also, those worms surviving treatment were noticeably smaller. In addition, the pathology normally associated with the deposition of schistosome eggs in the spleen and liver was clearly ameliorated. Finally, when extracts of surviving worms were tested for CP activity using selective substrates and active site labeling, K11777 had knocked down the activity of the major gut-associated protease, cathepsin B1 [31]. The identification of schistosome cathepsin B1 as a molecular target of K11777 is consistent with its central nutritive function in the digestion of host blood proteins [18,40] and RNA interference of its expression, although not lethal to cultured parasites [41,42], is nonetheless detrimental to worm growth and development [41].

Early treatment with K11777 effectively cured five of the seven mice treated—no eggs were found in the respective livers, due to the absence of female worms, and in the remaining two mice, egg production was negligible. Commensurate with these findings, spleen and liver pathologies, pronounced in untreated infected control mice, were not evident, and plasma ALT levels indicating liver function were close to normal. The significant amelioration of disease parameters upon early treatment was more dramatic than that seen with either the long course or the late treatments. This difference suggests that young parasites migrating through the skin and lungs are particularly sensitive to exogenous inhibitors, an observation that is consistent with previous studies of fluoromethyl ketone CP inhibitors [19]. Such effects on immature worms are drug specific, however. PZQ, for example, is effective against very young (aged <1 wk) invasive parasites in mice, but loses efficacy during the lung-migratory period and maturation of the parasites up to approximately 28 days p.i., at which time the drug regains potent bioactivity [5,6]. In contrast, the artemisinin derivatives (artemether and artesunate), which are being evaluated in combination with PZQ for therapy of schistosomiasis [6,43], are most active against the juvenile migratory stages and progressively lose bioactivity as parasites mature [6,44].

The greater sensitivity of young migratory S. mansoni to K11777 can be rationalized in terms of their containing less CP activity than older, larger worms that have reached the hepatic portal system. This hypothesis is supported by data reported from a study of larval parasites exposed to RNA interference targeting cathepsin B1 and subsequently injected into mice. Worms recovered 21 days later were markedly smaller than their counterparts exposed to off-target RNA interference [41]. It is only upon reaching the hepatic portal vessels (between days 8 and 14 p.i.) that cell division, blood feeding, and weight gain accelerate dramatically [28], and this elevated metabolism is positively correlated with increased specific CP activity [45]. Likewise, the greater sensitivity of male worms to K11777 in the late drug regimen can be explained by their containing less protease activity than females [46,47]. Females must ingest and degrade far greater amounts of blood proteins [48] in order to sustain the large-scale egg production characteristic of chronic schistosome infection.

An early treatment protocol would be ideal in cases where early diagnosis can be made, such as in tourists, aid workers, and military personnel. However, in endemic areas, most patients have established infections with mature egg-laying worms and concomitant hepatic complications. The late treatment protocol, begun at 30 days p.i. when worms have paired and commenced egg production [28,30], had a less dramatic effect on disease parameters compared to early drug treatment. Nonetheless, a striking decrease in egg and male worm burdens, smaller egg-associated granulomas, and decreased gross organ pathology were recorded. This is encouraging, both for preventing or retarding morbidity associated with the disease and for decreasing environmental contamination by schistosome eggs, and, thus, the level of disease transmission [1].

Because K11777 is in the late stages of preclinical development for treatment of Chagas' disease [20,21], it may be available for parallel clinical trials against schistosomiasis in the relatively near future. One intriguing possibility for its use would be in combination with PZQ (similar to that which has been proposed for derivatives of artemisinin), as the two compounds have very different mechanisms of action and could conceivably operate synergistically, as well as minimize the possibility of drug resistance.

## Abbreviations

ALT: alanine aminotransferase
BID: twice daily
CP: cysteine protease
i.p.: intraperitoneal(ly)
K11777: N-methylpiperazine-phenylalanyl-homophenylalanyl-vinylsulfone-phenyl
p.i.: postinfection
PZQ: praziquantel
